# Accuracy of Paediatric Advanced Weight Prediction in the Emergency Room-eXtra Length (PAWPER XL) Tape in Estimation of Body Weight in Pediatric Emergencies

**DOI:** 10.7759/cureus.89775

**Published:** 2025-08-11

**Authors:** Tejavath K Singh, Kafeel Khan, Suresh R J Thomas

**Affiliations:** 1 Pediatrics, Government General Hospital, Khammam, IND; 2 Pediatrics, Kamineni Institute of Medical Sciences, Nalgonda, IND

**Keywords:** anthropometry, body habitus, broselow tape, pawper xl tape, pediatric weight estimation

## Abstract

Background

Accurate weight estimation in pediatric emergencies is essential for safe drug dosing and equipment sizing. Traditional tools like the Broselow tape often lack precision in children with diverse body habitus. The Paediatric Advanced Weight Prediction in the Emergency Room - eXtra Length (PAWPER XL) tape, which incorporates both length and body habitus, has been proposed to improve estimation accuracy. This study aimed to evaluate the accuracy of the PAWPER XL tape compared to the Broselow tape among children admitted to a pediatric intensive care unit (PICU) in a resource-constrained setting.

Methods

A cross-sectional observational study was conducted at the PICU of Kamineni Institute of Medical Sciences, Narketpally, Telangana, from August 2022 to May 2024. One hundred children aged 3 months to 12 years were enrolled. Demographic and anthropometric data were recorded, and weight was estimated using both PAWPER XL and Broselow tapes. Accuracy was assessed using the proportion of estimates within 10% (PW10) and 20% (PW20) of actual weight, mean percentage error (MPE), and Bland-Altman 95% limits of agreement. Subgroup analyses were conducted based on weight categories and BMI-for-age Z-scores. McNemar’s test and Fisher’s exact test were used, with significance set at p<0.05.

Results

The PAWPER XL tape demonstrated significantly higher accuracy, with 83.5% of estimates within 10% (PW10) and 98.7% within 20% (PW20) of actual weight, compared to 61.2% and 87.9%, respectively, for the Broselow tape (χ² = 18.452, p < 0.001). PAWPER XL also showed lower bias (MPE: +1.2% vs. -1.6%) and narrower limits of agreement. Subgroup analysis confirmed its consistent accuracy across all weight and BMI-for-age categories, including underweight (PW10 = 69.4%), average (PW10 = 89.1%), and overweight (PW10 = 70.5%) children, while Broselow accuracy declined significantly in non-average habitus, especially overweight children (PW10 = 9.8%).

Conclusion

The PAWPER XL tape is a more accurate and clinically reliable tool for pediatric weight estimation in emergency settings compared to the Broselow tape. Its consistent performance across varying body types and its applicability in resource-limited settings like India underscore its value in improving pediatric emergency care outcomes.

## Introduction

In pediatric emergency care, accurately estimating a child’s body weight is vital for determining appropriate medication dosages, fluid resuscitation volumes, and defibrillation energy levels [1]. However, direct weighing is often impractical in critically ill or injured children due to time constraints, lack of equipment, or the urgency of clinical intervention. In such scenarios, healthcare providers must rely on weight estimation methods that combine speed, ease of use, and clinical accuracy [2].

The Broselow tape, introduced in the 1980s, is a length-based weight estimation tool that has been widely adopted in pediatric resuscitation. While its ease of use is well established, its accuracy is notably reduced in populations with a high prevalence of malnutrition or obesity - conditions that disrupt the standard weight-to-length ratio [3,4]. Multiple studies have reported that the Broselow tape often underestimates weight in overweight children and overestimates in undernourished children, with accuracy rates for predictions within 10% of actual weight (PW10) ranging only between 50% and 65% [5,6].

This limitation is particularly relevant in India, where children frequently present with wide anthropometric variability. According to the National Family Health Survey-5 (NFHS-5, 2019-21), 35.5% of children under five are stunted, 19.3% are wasted, and 3.4% are overweight, reflecting a dual burden of undernutrition and emerging childhood obesity [7,8]. Such extremes in nutritional status can lead to substantial inaccuracies in weight prediction using traditional length-only methods, increasing the risk of under- or overdosing during emergency care [9].

To overcome these limitations, the PAWPER XL (Paediatric Advanced Weight Prediction in the Emergency Room - eXtra Length) tape was developed. This tool incorporates both recumbent length and visual assessment of body habitus - scored from 1 (very underweight) to 7 (severely obese) - to improve weight estimation accuracy across a broader spectrum of body types [9,10]. Studies from high-income countries have demonstrated that the PAWPER XL tape can achieve weight estimates within 10% of actual weight in over 78% of cases, and within 20% in more than 96% [11].

Despite this evidence, the applicability and performance of the PAWPER XL tape remain underexplored in low- and middle-income countries like India, where nutritional diversity and resource constraints are prominent [12]. Limited Indian data suggest that length-plus-habitus methods outperform conventional tools, yet comprehensive validation studies, especially in emergency care settings, are still lacking [13]. Additionally, although the PAWPER XL tape offers clear advantages, its accurate use depends on correct habitus scoring, which may require brief training or standardization among healthcare workers - potentially posing a barrier to widespread adoption.

Given these considerations, this study aimed to evaluate the accuracy of the PAWPER XL tape in estimating body weight among Indian children admitted to a pediatric intensive care unit (PICU), using actual measured weight as the reference standard. By assessing its performance across diverse body types, this study seeks to establish its clinical utility and relevance in Indian emergency settings.

## Materials and methods

Study design and setting

This was a cross-sectional, observational study conducted in the PICU of the Department of Paediatrics at Kamineni Institute of Medical Sciences, Narketpally, Telangana. Data collection spanned from August 2022 to May 2024. The primary objective was to evaluate the accuracy of the PAWPER XL tape for weight estimation in critically ill pediatric patients and compare its performance against the Broselow tape, using actual weight measured by a calibrated digital scale as the reference standard.

Study population and sampling

A total of 100 children aged 3 months to 12 years, admitted consecutively to the PICU during the study period, were enrolled using purposive sampling. Inclusion criteria comprised hemodynamically stable children within the specified age range for whom recumbent length measurement was feasible. Exclusion criteria included: children below 3 months or above 12 years of age; those with limb deformities or contractures impeding length measurement; known syndromes affecting linear growth (Turner, Down, and Noonan syndromes); patients on long-term corticosteroid therapy; and those with significant dehydration or edema, which could distort true body weight. While purposive sampling ensured inclusion of a clinically relevant PICU cohort, this may limit generalizability beyond similar tertiary-care settings.

Measurement procedures

**Figure 1:**
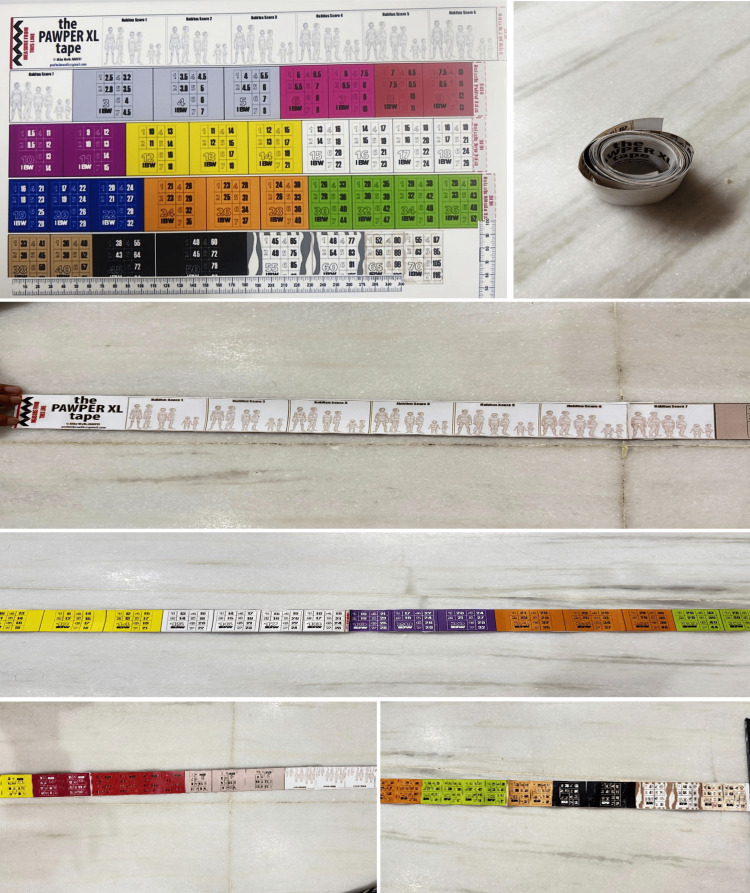
PAWPER XL on a paper These figures are the authors’ own work. PAWPER XL: Paediatric Advanced Weight Prediction in the Emergency Room - eXtra Length

Once clinically stable, each child was placed in a supine position on a flat surface. The PAWPER XL tape was first used to measure recumbent length from the crown to the heel. A visual gestalt assessment of body habitus was then performed using standardized reference images, assigning a habitus score (HS) from 1 to 7. The estimated weight was read from the segment of the tape corresponding to the child’s length and HS (Figure 1).

**Figure 2:**
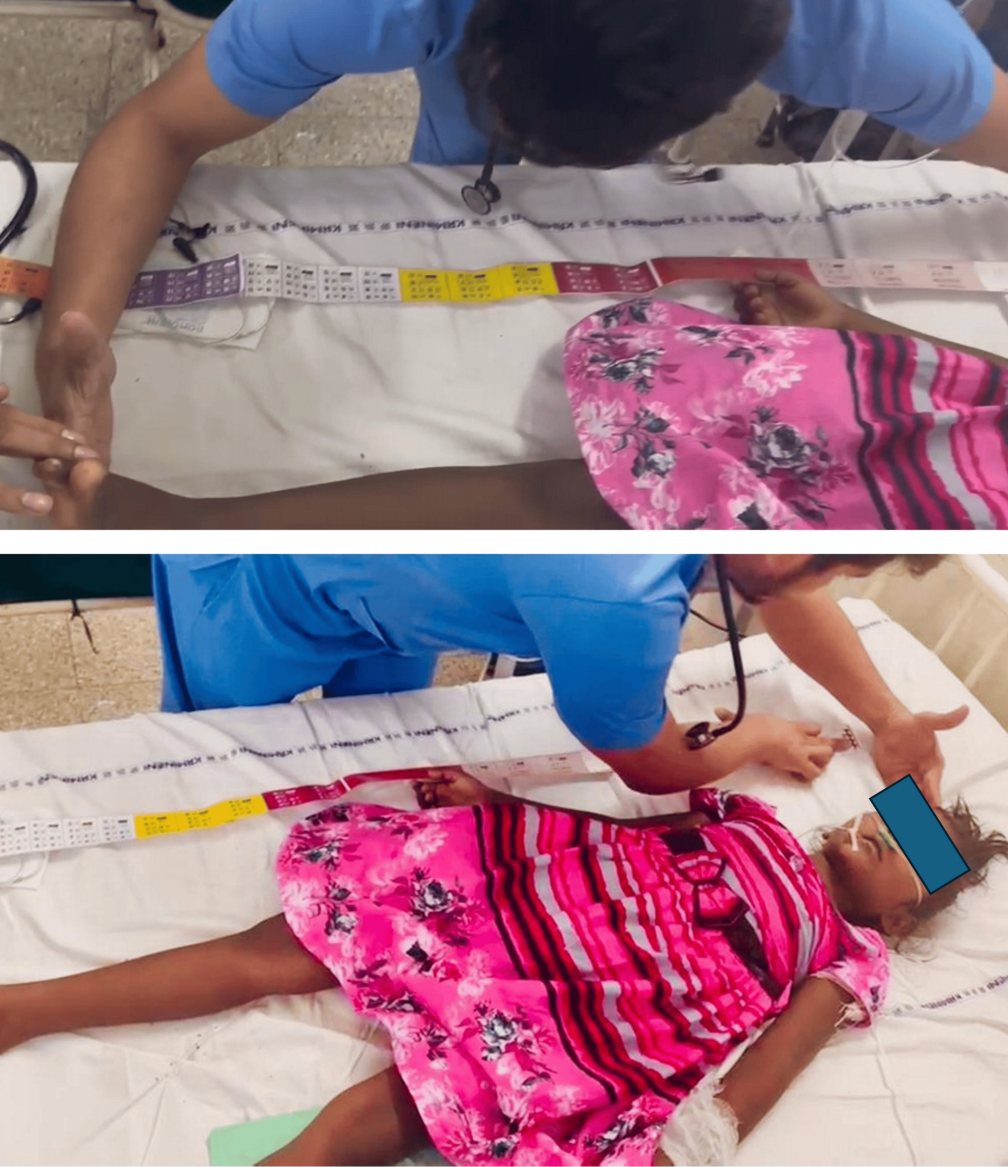
The use of the PAWPER XL tape on the patient PAWPER XL: Paediatric Advanced Weight Prediction in the Emergency Room - eXtra Length

Subsequently, the Broselow tape was used by aligning it similarly from head to heel, recording the estimated weight based on the color zone endpoint. The version used supports weight estimates up to 36 kg and serves as a conventional length-only comparator (Figure 2).

Actual weight determination

Actual weight (total body weight (TBW)) was determined using a calibrated electronic digital weighing scale with the child in minimal clothing and no accessories. Weighing was performed either during stabilization (when feasible) or post-recovery once clinically appropriate. The TBW was considered the gold standard for accuracy comparisons.

Data collection and categorization

Data collection was performed using a structured proforma, which captured each participant’s demographic details, clinical condition, actual measured weight, and the estimated weights obtained from both the PAWPER XL and Broselow tapes. To facilitate subgroup analyses, children were stratified into three actual weight categories: less than 10 kg, 10 to 25 kg, and greater than 25 kg. Additionally, the nutritional status of each child was categorized based on the World Health Organization’s BMI-for-age Z-score classification. Accordingly, children were classified as underweight if their Z-score was less than -2.0, of average weight if the Z-score ranged from -2.0 to +2.0, and overweight if the Z-score exceeded +2.0. This approach allowed for more nuanced evaluation of the accuracy of each weight estimation method across different body habitus types and nutritional strata, ensuring the results were reflective of anthropometric diversity.

Statistical analysis

All data were entered and analyzed using IBM SPSS Statistics version 20.0 (IBM Corp., Armonk, NY, USA). The accuracy of weight estimation methods was assessed using multiple parameters: the proportion of estimates falling within ±10% of actual weight (PW10), the proportion within ±20% (PW20), and the mean percentage error (MPE), which quantified the average directional bias of each method. To evaluate precision, Bland-Altman analysis was employed, and 95% limits of agreement around the MPE were calculated for both PAWPER XL and Broselow tapes. Accuracy levels were interpreted using predefined thresholds: PW10 values above 90% were considered exceptionally accurate, 70-90% as very high accuracy, 50-70% as moderate accuracy, 30-50% as low accuracy, and less than 30% as critically inaccurate. For PW20, values exceeding 95% denoted as extreme accuracy, 90-95% as high, 80-90% as moderate, and below 80% as indicative of a critical error rate. Comparative analyses between the PAWPER XL and Broselow tapes across various weight categories and BMI-based habitus groups were conducted using the Chi-square (χ²) test. Fisher’s exact test was applied when expected frequencies were less than five in any cell. Additionally, paired t-tests were used to compare the mean differences between actual and estimated weights. A p-value less than 0.05 was considered statistically significant for all analyses.

Ethical considerations

Ethical approval was obtained from the Institutional Ethics Committee of Kamineni Institute of Medical Sciences (Approval No: Ethics Committee/KIMS/NKP/Aug 2022/16; dated: 26/08/2022). Written informed consent was obtained from each child’s parent or legal guardian after a detailed explanation of the study procedures in their native language. The study followed the guidelines of the Declaration of Helsinki and adhered to institutional norms for research involving human participants.

## Results

The study included a total of 100 children, comprising 69 males (69%) and 31 females (31%). The majority of participants belonged to the 5-12 years age group (n = 48, 48%), followed by those aged 1-5 years (n = 33, 33%) and 3 months to 1 year (n = 19, 19%). Based on weight categories, 48 participants (48%) were in the 10-25 kg range, 30 participants (30%) weighed less than 10 kg, and 22 participants (22%) weighed more than 25 kg. According to BMI-for-age Z-scores, 76 children (76%) were in the normal range (-2.0 to +2.0), 13 (13%) were underweight (Z < -2.0), and 11 (11%) were overweight (Z > +2.0). Body composition assessed using the habitus score showed that 13 participants (13%) were classified as HS1-Underweight, 33 (33%) as HS2-thin, 29 (29%) as HS3-normal, 14 (14%) as HS4-slightly overweight, 10 (10%) as HS5-overweight, 1 (1%) as HS6-obese, and none (0%) as HS7-severely obese (Table 1).

**Table 1:** Demographic and Clinical Characteristics of Study Participants (n=100) HS: Habitus scores; Kgs: kilograms; BMI: body mass index

Variable	Category	Frequency	%
Age Group	3 months – 1 year	19	19
	1 – 5 years	33	33
	5 – 12 years	48	48
Sex	Male	69	69
	Female	31	31
Weight Category	<10 kgs	30	30
	10-25 kgs	48	48
	>25 kgs	22	22
BMI-for-age Z-score Group	Underweight (Z < -2.0)	13	13
	Normal (-2.0 to +2.0)	76	76
	Overweight (Z > +2.0)	11	11
Body Composition (Habitus Score)	HS1 – Underweight	13	13
	HS2 – Thin	33	33
	HS3 – Normal	29	29
	HS4 – Slightly overweight	14	14
	HS5 – Overweight	10	10
	HS6 – Obese	1	1
	HS7 – Severely obese	0	0

For the weight group <10 kg (n = 30, 30%), the mean actual weight was 8.3 ± 0.9 kg, while the PAWPER XL and Broselow tape estimated weights were 9.1 ± 1.0 kg and 10.4 ± 1.1 kg, respectively. The difference between PAWPER XL and actual weight was statistically significant (t = 3.622, p = 0.001), and the difference for the Broselow tape was highly significant (t = 9.458, p < 0.001). In the 10-25 kg group (n = 48, 48%), the mean actual weight was 16.8 ± 3.5 kg; estimates were 16.9 ± 3.2 kg for PAWPER XL (t = 0.164, p = 0.872) and 17.9 ± 3.6 kg for Broselow tape (t = 2.275, p = 0.028). For the >25 kg group (n = 22, 22%), the actual mean weight was 30.3 ± 4.2 kg, with PAWPER XL estimating 30.4 ± 4.5 kg (t = 0.101, p = 0.921), and Broselow tape estimating significantly lower at 25.1 ± 5.2 kg (t = 4.457, p < 0.001). Overall (n = 100), the mean actual weight was 17.8 ± 8.4 kg, while the PAWPER XL and Broselow tape estimates were 19.0 ± 8.5 kg (t = 2.044, p = 0.044) and 18.1 ± 7.6 kg (t = 1.208, p = 0.204), respectively (Table 2).

**Table 2:** Comparison of Actual Weight with Estimated Weights by PAWPER XL and Broselow Tape Kg: kilogram; PAWPER XL: Paediatric Advanced Weight Prediction in the Emergency Room - eXtra Length P-value<0.05 was considered statistically significant.

Group (Weight Range)	Actual Weight (kg)	PAWPER XL Estimate (kg)	Broselow Tape Estimate (kg)	PAWPER XL vs Actual (t, p-value)	Broselow Tape vs Actual (t, p-value)
	Mean ± SD				
<10 kgs (n=30)	8.3 ± 0.9	9.1 ± 1.0	10.4 ± 1.1	t = 3.622, p = 0.001	t = 9.458, p < 0.001
10-25 kgs (n=48)	16.8 ± 3.5	16.9 ± 3.2	17.9 ± 3.6	t = 0.164, p = 0.872	t = 2.275, p = 0.028
>25 kgs (n=22)	30.3 ± 4.2	30.4 ± 4.5	25.1 ± 5.2	t = 0.101, p = 0.921	t = 4.457, p < 0.001
Overall	17.8 ± 8.4	19.0 ± 8.5	18.1 ± 7.6	t = 2.044, p = 0.044	t = 1.208, p = 0.204

PAWPER XL demonstrated high accuracy across all weight categories. In the <10 kg group (n = 30, 30%), it showed moderate accuracy at PW10 (53.40%) and very high accuracy at PW20 (98.20%), with a statistically significant difference compared to the Broselow tape (χ² = 4.244, p = 0.039). In the 10-25 kg group (n = 48, 48%), PAWPER XL achieved very high accuracy at PW10 (84.90%) and extremely high accuracy at PW20 (99.80%), with a highly significant difference from Broselow tape (χ² = 11.687, p = 0.001). For the >25 kg group (n = 22, 22%), it maintained very high accuracy at both PW10 (84.60%) and PW20 (96.50%) (χ² = 10.323, p = 0.002). Overall (n = 100), PAWPER XL achieved very high accuracy at PW10 (83.50%) and extremely high accuracy at PW20 (98.70%) with a highly significant difference compared to the Broselow tape (χ² = 18.452, p < 0.001). In contrast, the Broselow tape showed lower performance. In the <10 kg group, it demonstrated good accuracy at PW10 (69.20%) and moderate accuracy at PW20 (79.50%). For 10-25 kg, accuracy was moderate at PW10 (63.90%) and high at PW20 (91.20%). In the >25 kg group, the Broselow tape showed low accuracy at PW10 (49.70%) and moderate accuracy at PW20 (81.40%). Overall, it achieved moderate accuracy at PW10 (61.20%) and high accuracy at PW20 (87.90%) (Table 3).

**Table 3:** Accuracy of PAWPER XL and Broselow Tape (PW10 and PW20 Analysis) Kg: Kilogram; PW10: percentage of weight estimates within ±10% of actual weight; PW20: percentage of weight estimates within ±20% of actual weight P-value<0.05 was considered statistically significant.

Method	Weight Category	PW10 (%)	PW20 (%)	PW10 Descriptor	PW20 Descriptor	χ², p-value (PAWPER vs Broselow)
PAWPER XL	<10 kg (n=30)	53.40%	98.20%	Moderate accuracy	Very high accuracy	χ² = 4.244, p = 0.039
	10–25 kg (n=48)	84.90%	99.80%	Very high accuracy	Extremely accurate	χ² = 11.687, p = 0.001
	>25 kg (n=22)	84.60%	96.50%	Very high accuracy	Very high accuracy	χ² = 10.323, p = 0.002
	Overall (n=100)	83.50%	98.70%	Very high accuracy	Extremely accurate	χ² = 18.452, p < 0.001
Broselow Tape	<10 kg (n=30)	69.20%	79.50%	Good accuracy	Moderate accuracy	—
	10–25 kg (n=48)	63.90%	91.20%	Moderate accuracy	High accuracy	—
	>25 kg (n=22)	49.70%	81.40%	Low accuracy	Moderate accuracy	—
	Overall (n=100)	61.20%	87.90%	Moderate accuracy	High accuracy	—

PAWPER XL demonstrated excellent accuracy across all BMI categories, with high accuracy PAWPER XL demonstrated excellent accuracy across all BMI-for-age Z-score categories. In the thin group (n = 13, 13%), it achieved moderate accuracy at PW10 (69.40%) and high accuracy at PW20 (98.60%), with significantly better performance than the Broselow tape (χ² = 5.913, p = 0.015). In contrast, the Broselow tape showed poor accuracy in this group (PW10 = 21.50%, PW20 = 58.60%). Among children with average BMI-for-age (n = 76, 76%), PAWPER XL showed very high accuracy at PW10 (89.10%) and nearly perfect accuracy at PW20 (99.50%), again with a statistically significant difference from the Broselow tape (χ² = 4.843, p = 0.028), which had moderate accuracy at PW10 (76.20%) and high accuracy at PW20 (98.50%). In the overweight group (n = 11, 11%), PAWPER XL showed good accuracy at PW10 (70.50%) and very high accuracy at PW20 (92.90%), while the Broselow tape performed very poorly (PW10 = 9.80%, PW20 = 58.60%), with a significant difference in accuracy (χ² = 8.122, p = 0.004). Overall (N = 100), PAWPER XL achieved high accuracy at PW10 (83.50%) and extremely high accuracy at PW20 (98.70%), significantly outperforming the Broselow tape (PW10 = 61.20%, PW20 = 87.90%) (χ² = 10.335, p = 0.001) (Table 4).

**Table 4:** Subgroup Analysis of Accuracy by BMI-for-Age Z-score (Habitus Score Based) Kg: Kilogram; PW10: percentage of weight estimates within ±10% of actual weight; PW20: percentage of weight estimates within ±20% of actual weight; MPE: Mean Percentage Error: BMI: Body Mass Index; Thin =1,2 of Habitus Score, Average=3 of Habitus Score, Overweight= 4,5,6 of Habitus Score; PAWPER XL: Paediatric Advanced Weight Prediction in the Emergency Room - eXtra Length P-value<0.05 was considered statistically significant.

BMI Z-score Category	Method	PW10 (%)	PW20 (%)	MPE (%)	95% CI	χ², p-value
Thin	PAWPER XL	69.40%	98.60%	6.5	-2.4 to 17.5	χ² = 5.913, p = 0.015
	Broselow Tape	21.50%	58.60%	-5.2	-29.5 to 19.7	
Average	PAWPER XL	89.10%	99.50%	1.6	-11.4 to 12.1	χ² = 4.843, p = 0.028
	Broselow Tape	76.20%	98.50%	-1.9	-18.4 to 16.1	
Overweight	PAWPER XL	70.50%	92.90%	1.9	-15.9 to 9.5	χ² = 8.122, p = 0.004
	Broselow Tape	9.80%	58.60%	-18.8	-28.1 to 4.9	
All Categories	PAWPER XL	83.50%	98.70%	1.2	-12.9 to 15.2	χ² = 10.335, p = 0.001
	Broselow Tape	61.20%	87.90%	-1.6	-18.65 to 23.1	

## Discussion

Accurate weight estimation remains a cornerstone in pediatric emergency medicine, as weight directly determines appropriate drug dosing, fluid therapy, and equipment sizing [13]. Our study, conducted in the PICU at Kamineni Institute of Medical Sciences, Telangana, on 100 children aged 3 months to 12 years, adds to the growing body of evidence supporting the superiority of the PAWPER XL tape over traditional tools like the Broselow tape. The PAWPER XL consistently demonstrated higher accuracy in weight estimation across varied weight categories and BMI-for-age Z-score habitus classifications.

The demographic distribution revealed a male predominance (n=69, 69%), aligning with prior studies by Ong and Dy and Silvagni et al., where boys represented nearly two-thirds of the cohorts [14,15]. However, contrasting findings from Indian research by Setlur et al., Asskaryar and Shankar, and Varghese et al. indicated a more balanced gender distribution [16-18]. These discrepancies could reflect local referral patterns or sociocultural variations in healthcare-seeking behaviors.

The Broselow tape’s declining accuracy with increasing child size and altered habitus has been well-documented. Iloh et al. reported significant underestimation in older children, particularly beyond 5 years of age [19]. In our study, this was reflected in the >25 kg weight category, where Broselow estimated an average weight of 25.1 ± 5.2 kg, significantly lower than the actual average of 30.3 ± 4.2 kg (t = 4.457, p < 0.001). In contrast, PAWPER XL produced a near-identical estimate (30.4 ± 4.5 kg; t = 0.101, p = 0.921), demonstrating minimal bias. This observation reinforces the growing consensus that length-only models fail in populations with high nutritional variability [20].

Overall, PAWPER XL achieved PW10 and PW20 accuracies of 83.5% and 98.7%, respectively, substantially outperforming Broselow (PW10 = 61.2%, PW20 = 87.9%) with a highly significant difference (χ² = 18.452, p < 0.001). These findings echo results from Foster et al. and Wells et al., who reported that the Broselow tape underperformed, particularly in children with extreme body types [21,22]. Our results also support Manyoni et al., who identified PAWPER XL as the most accurate among four tested weight estimation tools, achieving a PW10 of 74% [23].

Subgroup analysis further validated PAWPER XL’s robustness. In children weighing <10 kg (n=30), it recorded a modest PW10 of 53.4% but a very high PW20 of 98.2% (χ² = 4.244, p = 0.039), indicating acceptable performance even in infants. Accuracy improved markedly in older children: in the 10-25 kg (n=48) and >25 kg (n=22) categories, PW10 rose to 84.9% and 84.6%, respectively. The Broselow tape underperformed in all these subgroups, with its lowest performance seen in overweight children (PW10 = 49.7%; PW20 = 81.4%).

BMI-for-age stratification revealed the PAWPER XL’s adaptability to varied nutritional statuses. Among thin children (n=20), PW10 was 69.4%, PW20 98.6%, and MPE 6.5% (χ² = 5.913, p = 0.015). For average-BMI children (n=60), the tape performed exceptionally with PW10 = 89.1%, PW20 = 99.5%, and MPE 1.6% (χ² = 4.843, p = 0.028). Overweight children (n=20) also saw good performance with PW10 = 70.5%, PW20 = 92.9%, and MPE 1.9% (χ² = 8.122, p = 0.004). The Broselow tape’s accuracy was particularly poor in non-average body types: PW10 was only 21.5% in thin children and 9.8% in overweight children. This sharp contrast highlights Broselow’s inherent limitation in one-dimensional estimation.

These patterns mirror findings from international studies such as those by Cosmos Yakubu et al., and Shrestha et al., which also found the PAWPER XL superior in both precision and accuracy across habitus types [24,25]. In the Indian context, Asskaryar and Shankar proposed a new tool, the Indian Pediatric Emergency Weight Estimation Tool (IPEWET), acknowledging that the Broselow tape consistently overestimated weights in Indian children [17]. Similarly, Varghese et al. cautioned against using Broselow beyond the age of 6 or in children weighing more than 15 kg [18], again emphasizing the need for tools that adjust for body composition.

Importantly, the PAWPER XL tape not only estimates TBW but also ideal body weight (IBW), which has significant implications for pediatric resuscitation. Wells et al. highlighted the risks of overdosing hydrophilic drugs when using TBW in obese children and underdosing when using IBW in underweight children [21]. The PAWPER XL tape, by integrating both parameters, addresses this dilemma and enhances dosing safety [26,27]. In settings like India, where both undernutrition and pediatric obesity coexist, this dual functionality is particularly advantageous.

A notable strength of our study lies in the use of BMI-for-age Z-scores for categorizing habitus, which aligns with WHO growth standards and allows international comparability. Furthermore, our study is among the few Indian investigations that have examined PAWPER XL’s accuracy with detailed subgroup analyses, enhancing its relevance for local practice. The rigorous methodology, including standardized measurement protocols, use of calibrated digital weighing scales, and application of appropriate statistical tools, lends credibility to our findings.

Limitations

However, several limitations must be acknowledged. First, the sample size, though adequate for initial analysis, was limited to a single tertiary-care center, potentially limiting generalizability. Second, the severely obese category (HS7) was underrepresented (n=1), precluding meaningful subgroup analysis for this habitus score. Future multicentric studies with larger sample sizes and a broader range of habitus types are essential. Third, the accuracy of PAWPER XL habitus scoring depends on the clinician’s subjective visual assessment. Although all investigators were trained using standardized visual references, interobserver variability was not evaluated. Incorporating digital or photographic tools in future studies could help reduce subjectivity and enhance reproducibility. Additionally, the cross-sectional design limits the ability to assess learning curves or the impact of repeated usage on observer accuracy.

Despite these limitations, our study supports the adoption of PAWPER XL tape in pediatric emergency settings, particularly in resource-limited environments like India, where rapid, reliable, and safe weight estimation is critical. The tool’s high accuracy across a range of body types and its relevance to Indian anthropometric profiles make it a valuable asset in pediatric emergency care protocols.

## Conclusions

The PAWPER XL tape demonstrated significantly greater accuracy than the Broselow tape for pediatric weight estimation across all weight categories and body habitus types. With over 83% of estimates falling within 10% of actual weight (PW10), the PAWPER XL proved to be a highly reliable tool, particularly in populations where traditional length-based methods, like the Broselow tape, often fail-such as in underweight or overweight children. Its dual-parameter approach, incorporating both length and body habitus, enhances its adaptability to diverse pediatric populations, making it especially suitable for resource-limited and nutritionally heterogeneous settings like India. Incorporation of the PAWPER XL tape into pediatric emergency protocols could lead to more accurate drug and fluid dosing, thereby improving patient safety and treatment outcomes. Nonetheless, further multicentric research involving larger and more diverse cohorts, including severely obese children and varied clinical scenarios, is warranted to validate its broader applicability and refine implementation strategies in routine practice.

## References

[1] Partridge RL, Abramo TJ, Haggarty KA, Hearn R, Sutton KL, An AQ, Givens TG (2009). Analysis of parental and nurse weight estimates of children in the pediatric emergency department. Pediatr Emerg Care.

[2] Young KD, Korotzer NC (2016). Weight estimation methods in children: A systematic review. Ann Emerg Med.

[3] Knight JC, Nazim M, Riggs D, Channel J, Mullet C, Vaughan R, Wilson A (2011). Is the Broselow tape a reliable indicator for use in all pediatric trauma patients?: A look at a rural trauma center. Pediatr Emerg Care.

[4] Pukar KC, Jha A, Ghimire K, Shrestha R, Shrestha AP (2020). Accuracy of Broselow tape in estimating the weight of the child for management of pediatric emergencies in Nepalese population. Int J Emerg Med.

[5] Lowe CG, Campwala RT, Ziv N, Wang VJ (2016). The Broselow and Handtevy resuscitation tapes: A comparison of the performance of pediatric weight prediction. Prehosp Disaster Med.

[6] Zhu S, Zhu J, Zhou H (2022). Validity of Broselow tape for estimating the weight of children in pediatric emergency: A cross-sectional study. Front Pediatr.

[7] Soni J, Sheikh FS, Saha S, Wanjari MB, Saxena D (2023). Nutritional indicators for Gujarat, its determinants and recommendations: A comparative study of National Family Health Survey-4 and National Family Health Survey-5. Cureus.

[8] Jha A, Chandrakar A (2024). A comparative study of National Family Health Survey-4 and National Family Health Survey-5 of nutritional indicators in Chhattisgarh. Cureus.

[9] Wells JC, Sawaya AL, Wibaek R, Mwangome M, Poullas MS, Yajnik CS, Demaio A (2020). The double burden of malnutrition: Aetiological pathways and consequences for health. Lancet.

[10] Wells M, Goldstein LN, Bentley A (2017). Development and validation of a method to estimate body weight in critically ill children using length and mid-arm circumference measurements: The PAWPER XL-MAC system. S Afr Med J.

[11] Wu MT, Wells M (2020). Pediatric weight estimation: Validation of the PAWPER XL tape and the PAWPER XL tape mid-arm circumference method in a South African hospital. Clin Exp Emerg Med.

[12] Georgoulas VG, Wells M (2016). The PAWPER tape and the Mercy method outperform other methods of weight estimation in children at a public hospital in South Africa. S Afr Med J.

[13] Wells M (2019). A validation of the PAWPER XL-MAC tape for total body weight estimation in preschool children from low- and middle-income countries. PLoS One.

[14] Ong GJ, Dy E (2020). Validation of two pediatric resuscitation tapes. J Am Coll Emerg Physicians Open.

[15] Silvagni D, Baggio L, Mazzi C, Cuffaro G, Carlassara S, Spada S, Biban P (2022). The PAWPER tape as a tool for rapid weight assessment in a Paediatric Emergency Department: Validation study and comparison with parents' estimation and Broselow tape. Resusc Plus.

[16] Setlur K, Sankar J, Kapil U, Pandey RM, Kabra SK, Lodha R (2024). Development and validation of a weight estimation tool for acutely ill children who cannot be weighed. Indian J Pediatr.

[17] Asskaryar F, Shankar R (2015). An Indian pediatric emergency weight estimation tool: Prospective adjustment of the Broselow tape. Int J Emerg Med.

[18] Varghese A, Vasudevan VK, Lewin S, Indumathi CK, Dinakar C, Rao SD (2006). Do the length-based (Broselow) Tape, APLS, Argall and Nelson's formulae accurately estimate weight of Indian children?. Indian Pediatr.

[19] Iloh ON, Edelu B, Iloh KK (2019). Weight estimation in Paediatrics: How accurate is the Broselow-tape weight estimation in the Nigerian child. Ital J Pediatr.

[20] Choi S, Nah S, Kim S, Seong EO, Kim SH, Han S (2022). A validation of newly developed weight estimating tape for Korean pediatric patients. PLoS One.

[21] Foster M, Tagg A, Klim S, Kelly AM (2019). Accuracy of parental estimate of child's weight in a paediatric emergency department. Emerg Med Australas.

[22] Wells M, Goldstein LN, Bentley A (2017). It is time to abandon age-based emergency weight estimation in children! A failed validation of 20 different age-based formulas. Resuscitation.

[23] Manyoni MJ, Goldstein LN, Wells M (2019). A comparison of four weight estimation systems for paediatric resuscitation. S Afr J Surg.

[24] Cosmos Yakubu R, Ayi-Bisah N, Nguah SB (2022). Accuracy of weight estimation in children using the Broselow, PAWPER XL, PAWPER XL-MAC, and Mercy Tapes. Pediatr Emerg Care.

[25] Shrestha K, Subedi P, Pandey O, Shakya L, Chhetri K, House DR (2018). Estimating the weight of children in Nepal by Broselow, PAWPER XL and Mercy method. World J Emerg Med.

[26] O'Leary F, John-Denny B, McGarvey K, Hann A, Pegiazoglou I, Peat J (2017). Estimating the weight of ethnically diverse children attending an Australian emergency department: A prospective, blinded, comparison of age-based and length-based tools including Mercy, PAWPER and Broselow. Arch Dis Child.

[27] Ralston ME, Myatt MA (2018). Weight estimation for children aged 6 to 59 months in limited-resource settings: A proposal for a tape using height and mid-upper arm circumference. PLoS One.

